# Application of metagenomic next-generation sequencing in patients with infective endocarditis

**DOI:** 10.3389/fcimb.2023.1107170

**Published:** 2023-02-03

**Authors:** Shao-Lin Li, Xi Zhao, Jun-Zhong Tao, Zhen-Zhen Yue, Xiao-Yan Zhao

**Affiliations:** Department of Cardiology, Cardiovascular Center, Henan Key Laboratory of Hereditary Cardiovascular Diseases, The First Affiliated Hospital of Zhengzhou University, Zhengzhou, Henan, China

**Keywords:** metagenomic next-generation sequencing, infective endocarditis, culture-negative, culture-positive, antibiotic regimen

## Abstract

**Objectives:**

Metagenomic next-generation sequencing (mNGS) technology is helpful for the early diagnosis of infective endocarditis, especially culture-negative infective endocarditis, which may guide clinical treatment. The purpose of this study was to compare the presence of culture-negative infective endocarditis pathogens versus culture-positive ones, and whether mNGS test results could influence treatment regimens for patients with routine culture-negative infective endocarditis.

**Methods:**

The present study enrolled patients diagnosed with infective endocarditis and tested for mNGS in the First Affiliated Hospital of Zhengzhou University from February 2019 to February 2022 continuously. According to the culture results, patients were divided into culture-negative group (Group CN, n=18) and culture-positive group (Group CP, n=32). The baseline characteristics, clinical data, pathogens, 30 day mortality and treatment regimen of 50 patients with infective endocarditis were recorded and analyzed.

**Results:**

Except for higher levels of PCT in the Group CN [0.33 (0.16-2.74) ng/ml *vs*. 0.23 (0.12-0.49) ng/ml, P=0.042], there were no significant differences in the basic clinical data and laboratory examinations between the two groups (all P>0.05). The aortic valve and mitral valve were the most involved valves in patients with infective endocarditis (aortic valve involved: Group CN 10, Group CP 16; mitral valve involved: Group CN 8, Group CP 21; P>0.05) while 9 patients had multiple valves involved (Group CN 2, Group CP 7; P>0.05). The detection rate of non-streptococci infections in the Group CN was significantly higher than that in the Group CP (9/18 *vs*. 3/32, P=0.004). There was no significant difference in patients with heart failure hospitalization and all-cause death at 30 days after discharge (3 in Group CN *vs*. 4 in Group CP, P>0.05). It is worth noting that 10 patients with culture-negative infective endocarditis had their antibiotic regimen optimized after the blood mNGS.

**Conclusions:**

Culture-negative infective endocarditis should be tested for mNGS for early diagnosis and to guide clinical antibiotic regimen.

## Introduction

Infective endocarditis (IE) is an inflammation disease with high morbidity and mortality (Thuny et al., 2012; Toyoda et al., 2017). The prognosis of IE patients depends on the accurate diagnosis and timely treatment, while rapid and precise pathogen identification is a key point for the etiological diagnosis of IE and guides antibiotic choice and adjustment (Cahill et al., 2017; Iung and Duval, 2019). Blood culture is currently the standard method for IE diagnosis, but routine blood culture for IE has low sensitivity, mainly due to previous antibiotic treatment and microorganisms being fastidious or unculturable. The incidence of blood culture negative endocarditis has been reported to vary from 15% to 60% (Letaief et al., 2007; Kadri et al., 2019; Shah et al., 2020). The high false negative rate, long blood culture time, and delay in timely diagnosis and treatment significantly adversely affect the prognosis. Therefore, there is an urgent need to improve the accuracy and effectiveness of diagnosis in order to reduce the mortality of IE patients through early intervention.

Recently, metagenomic next-generation sequencing (mNGS) has been applied to pathogen detection in infectious diseases as a universal method to sequence and identify nucleic acids from microorganisms. In addition to high efficiency and accuracy, the advantages of mNGS include non-culture-based. Compared to the conventional approach, mNGS is known for its comprehensive and unbiased nature with the capability to detect any potential pathogen in clinical samples, especially in the detection of viruses, fungi, and anaerobic bacteria. Currently, few studies have reported on the application of mNGS in IE. Studies have shown that valve mNGS of blood culture negative IE was helpful in the detection of uncommon pathogens such as the bacteria of the HACEK group (Lützen et al., 2018), and can improve clinical prognosis (Fournier et al., 2010; Miao et al., 2018; Gu et al., 2021). However, the application of valve mNGS in non-surgical IE patients is limited, and is not facilitated to control the infection as early as possible. Therefore, our study was aimed to discuss the value of blood mNGS in optimizing IE antibiotic treatment during early clinical practice.

## Materials and methods

### Patients and study design

**Figure 1:**
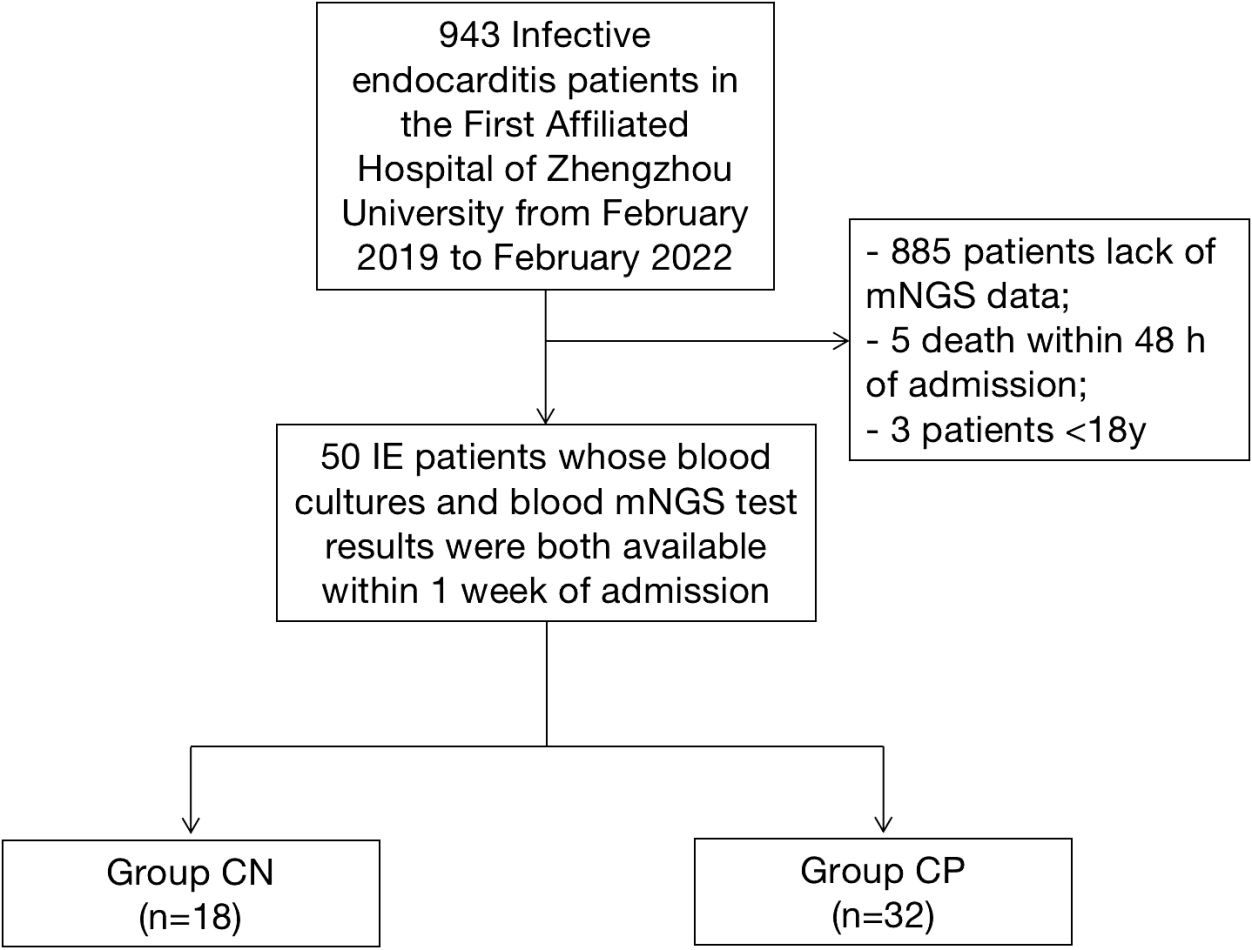
Flow Chart.

The present study enrolled IE patients whose blood cultures and blood mNGS test results were both available within 1 week of admission in the First Affiliated Hospital of Zhengzhou University from February 2019 to February 2022 continuously. The modified Duke criteria including etiological results were used to diagnosis of definite IE (Li et al., 2000). The exclusion criteria were as follows: 1) aged<18 years old; 2) death occurred within 48 h of admission; 3) patients who did not agree to written consent for mNGS examination; 4) missing informed consent. Finally, 50 IE patients who have both blood cultures and blood mNGS test results were included in the study (**Figure 1**). According to the blood culture results, patients were divided into culture-negative group (Group CN, n=18) and culture-positive group (Group CP, n=32). The baseline characteristics, clinical data, pathogens, 30 day mortality and treatment regimen of 50 patients with infective endocarditis were recorded and analyzed. The clinical data of the IE patients were obtained through the electronic medical record system. The endpoint were heart failure hospitalization and all-cause death at 30 days after discharge.

The present study was approved by the Ethics Committee of the First Affiliated Hospital of Zhengzhou University, Zhengzhou, China (no. 2022-KY-274). All procedures performed in this study involving human participants were in accordance with the Declaration of Helsinki. All participants provided written informed consent before participation.

### Blood culture

All included IE patients underwent blood culture examination after admission. Blood culture refers to the ESC Guidelines for the management of IE. Each patient received 3 successive sets of blood culture by peripheral venous puncture after admission. Each culture bottle (including aerobic bottles and anaerobic bottles) (THERMO SCIENTIFIC, Remel. Inc, Kansas, USA) with 10 ml of blood was sent to the clinical microbiology room for culture within 30 min, and then loaded into an automated continuous monitoring system for 7 days. And the blood samples were also sent for mNGS testing at the same time.

### Blood mNGS

The whole blood samples were collected from IE patients in the mean time with blood culture, and then about 300ul plasma (the whole blood sample centrifugated at 4°C) of each patient was used to extracted DNA by TIANamp Micro DNA Kit following the manufacturer’s instructions. DNA libraries were quality controlled using an Agilent 2100 Bioanalyzer (Agilent, USA) and an ABI StepOnePlus real-time polymerase chain reaction (PCR) system, and qualified libraries were sequenced on a NextSeq 550Dx platform (illumina, USA) using a 75 bp sequencing read length as previously described (Zhao et al., 2022). After the low-quality reads were removed, the clean reads were aligned to human genome to remove host sequence reads. All selected reads were mapped to the commercial pathogen database to annotate and analyze. Template free control (NTC) was obtained by repeated DNA extraction and sequencing using blank tubes filled with UltraPure Dnase/Rnase-free distilled water.

The data that support the findings of this study have been deposited into EMBL database with accession number PRJEB57970.

### Statistical analysis

Continuous variables were expressed as mean ± standard deviation (SD) or median (interquartile 25-75) as appropriate, and comparative analysis between two groups was conducted by Student’s t or rank-sum test (Mann-Whitney U). Categorical variables were analyzed using the Chi-square test for trend or Fisher’s exact test. The statistical software SPSS version 21.0 (IBM Corp., Chicago, IL, USA) was used for data analysis. Two-sided p value <0.05 was considered to be of statistical significance.

## Results

### Clinical characteristics and short-term outcomes

A total of 50 IE patients were included in the present study, among which 18 were blood culture-negative IE patients and 32 were blood culture-positive IE cases. Approximately 90% (16/18, 88.9%, **Table 1**) of blood culture-negative IE patients were males, with an average age of 48 ± 15.8 years and an average BMI of 23.5 ± 4.0 kg/m^2^. Similar to the Group CN, the blood culture-positive IE group was consisted by 23 males (71.9%), with an average age of 46 ± 15 years and an average BMI of 23.8 ± 1.9 kg/m^2^. In the CN group, there were 2 (11.1%) previous cardiac surgery cases, 12 (66.7%) antibiotics used before admission cases, 2 (11.1%) cerebral embolism cases, and 3 (16.7%) cases occurred events during 30 days follow up (1 death and 2 heart failure hospitalization). In the CP group, there were 15 (46.9%) cases of antibiotics used before admission, 9 (28.1%) cases of organ embolism (8 cerebral embolism and 1 splenic embolism), and 4 (12.5%) cases of events (1 death and 3 heart failure hospitalization).

**Table 1:** Clinical characteristics of culture-negative IE and culture-positive IE patients.

Characteristic	Group CN (n=18)	Group CP (n=32)	P-value
**Male**	16 (88.9%)	23 (71.9%)	0.132
**Age** (y)	48 ± 15.8	46 ± 15.0	0.710
**BMI** (Kg/M^2^)	23.5 ± 4.0	23.8 ± 1.9	0.748
Comorbidities			
Hypertension	3 (16.7%)	9 (28.1%)	0.373
Type 2 diabetes	2 (11.1%)	1 (3.1%)	0.263
Previous cardiac surgery	2 (11.1%)	0 (0%)	0.163
Clinical features			
Body temperature at admission	37.0 ± 0.9	36.7 ± 0.6	0.160
Fever (after admission)	12 (66.7%)	21 (65.6%)	0.942
The maximum temperature after admission	38.4 ± 1.3	38.2 ± 1.4	0.607
Antibiotics used before admission	12 (66.7%)	15 (46.9%)	0.185
Organ embolism	2 (11.1%)	9 (28.1%)	0.132
Surgical valve surgery	12 (66.7%)	27 (84.3%)	0.172
**Events**	3 (16.7%)	4 (12.5%)	0.692

The data was shown as the mean ± SD or n (percentage). IE, infective endocarditis; BMI, body mass index.

As shown in **Table 2**, the levels of PCT was higher in CN patients than that in CP cases (0.33 (0.16-2.74) ng/mL *vs*. 0.23 (0.12-0.49) ng/mL, p=0.042, **Table 2**). The levels of other laboratory examinations such as ESR, CRP, NT-proBNP in the CN and CP groups have shown no statistical differences (all p>0.05, **Table 2**). Echocardiogram manifestations of IE patients were shown in **Table 3**. The most involved valves of IE were aortic valve and mitral valve, and valvular vegetations could be present in two valves (Group CN: 2/11.1%; Group CP: 7/21.9%). Moreover, the complications of IE such as valve perforation and pericardial effusion also occur in CN and CP patients. However, there was no significant difference between the CN and CP patients in the clinical characteristics, Laboratory data, echocardiogram manifestation, and events except for the levels of PCT.

**Table 2:** Compared of laboratory examinations in culture-negative IE and culture-positive IE patients.

Laboratory data	Group CN (n=18)	Group CP (n=32)	P-value
**WBC**, (10^9^/L)	9.9 (6.4-12.7)	7.6 (6-10.3)	0.175
**Percentage of neutrophils**, (%)	76 ± 13.6	77.5 ± 14.7	0.727
**ESR**, (mm/H)	50 ± 32.6	45 ± 26	0.595
**CRP**, (mg/L)	62.5 ± 43.4	55.8 ± 40.9	0.616
PCT*, (ng/mL)	0.33 (0.16-2.74)	0.23 (0.12-0.49)	**0.042**
**Hemoglobin**, (g/L)	110.6 ± 20.1	105.1 ± 18	0.326
**Platelet**, (10^9^/L)	178 ± 87.4	187 ± 71.2	0.683
**Creatinine**, (umol/L)	93.1 ± 58.3	78.9 ± 38.5	0.306
**Total bilirubin**, (umol/L)	66.5 ± 8.3	65.3 ± 5.8	0.574
**CKMB**, (U/L)	11 (5-15)	8 (5-14)	0.171
**NT-proBNP**, (pg/mL)	455 (198-2084)	443 (239-1161)	0.352

The data was shown as the mean ± SD or median (interquartile 25-75). * indicate statistical significance. IE, infective endocarditis; WBC, white blood cell; ESR, erythrocyte sedimentation rate; CRP, C-Reactive protein; PCT, procalcitonin; CKMB, creatine kinase-MB; NT-proBNP, N-terminal pro-B-type natriuretic peptide. The meaning of the bold value is statistical significance (p<0.05).

**Table 3:** Echocardiogram manifestations of the culture-negative IE and culture-positive IE patients.

Echocardiogram manifestation	Group CN (n=18)	Group CP (n=32)	P-value
Valvular vegetations			
Aortic valve	10 (55.6%)	16 (50%)	0.713
Mitral valve	8 (44.4%)	21 (65.6%)	0.151
Tricuspid valve	2 (11.1%)	1 (3.1%)	0.342
Pulmonary valve	0 (0%)	1 (3.1%)	0.459
**Clinical features**			0.317
1 valve	16 (88.9%)	25 (78.1%)	
2 valves	2 (11.1%)	7 (21.9%)	
**Perforation**	2 (11.1%)	5 (15.6%)	0.667
**Pericardial effusion**	1 (5.6%)	4 (12.5%)	0.538
**LVED**, (mm)	53.9 ± 7.2	57.3 ± 8.8	0.171
**LVEF**, (%)	61.2 ± 7.9	60.8 ± 5.9	0.848
**Cross sectional area of vegetations**, (mm^2^)	82 (44.6-126)	68.8 (37.1-217.8)	0.435

The data was shown as the mean ± SD, median (interquartile 25-75) or n (percentage). IE, infective endocarditis; LVED, left ventricular end-diastolic diameter; LVEF, left ventricular ejection fraction.

### Pathogens detected by mNGS

**Figure 2:**
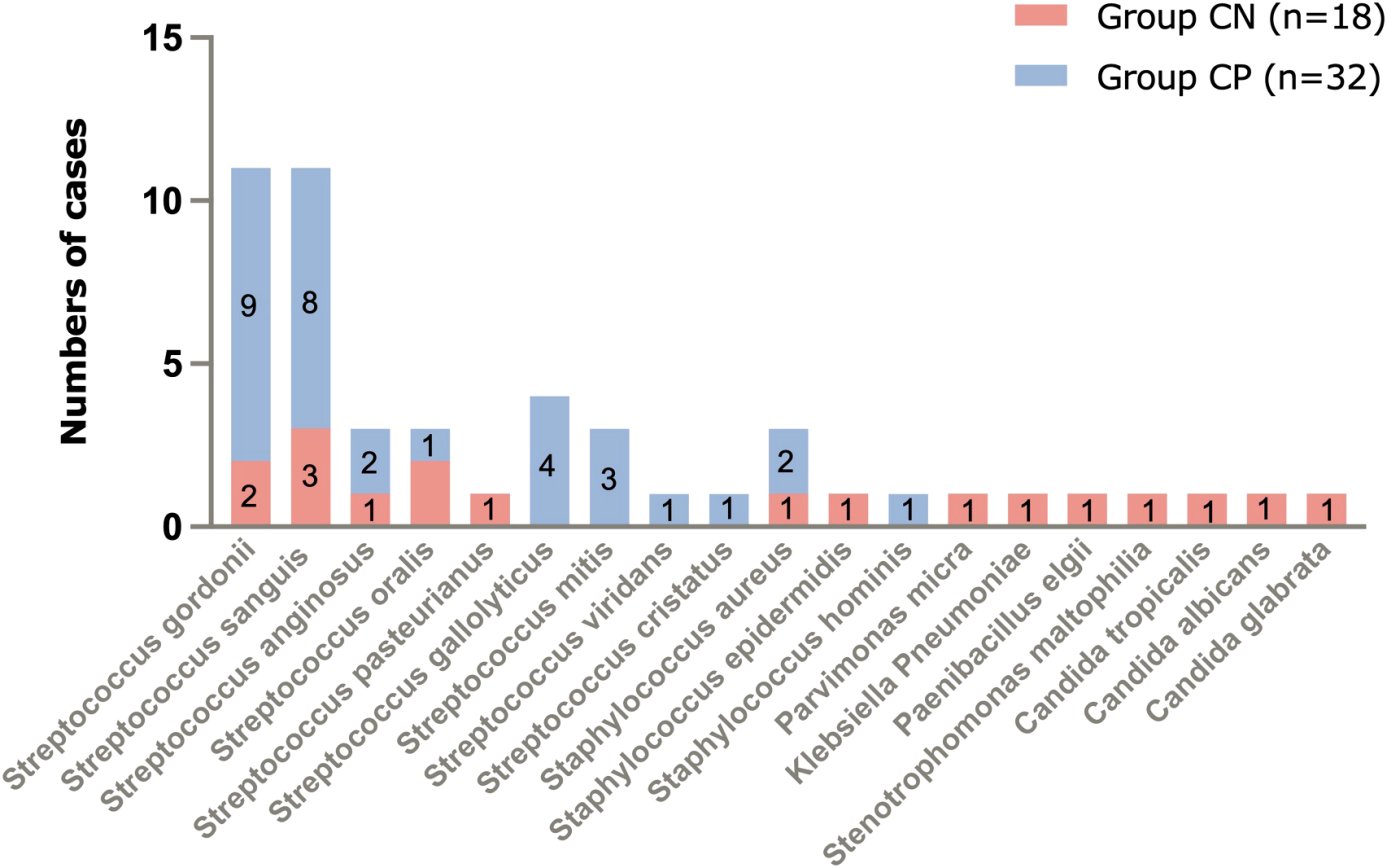
Pathogens of IE detected by blood mNGS and number of cases. mNGS, metagenomic next-generation sequencing; IE, infective endocarditis.

**Figure 3:**
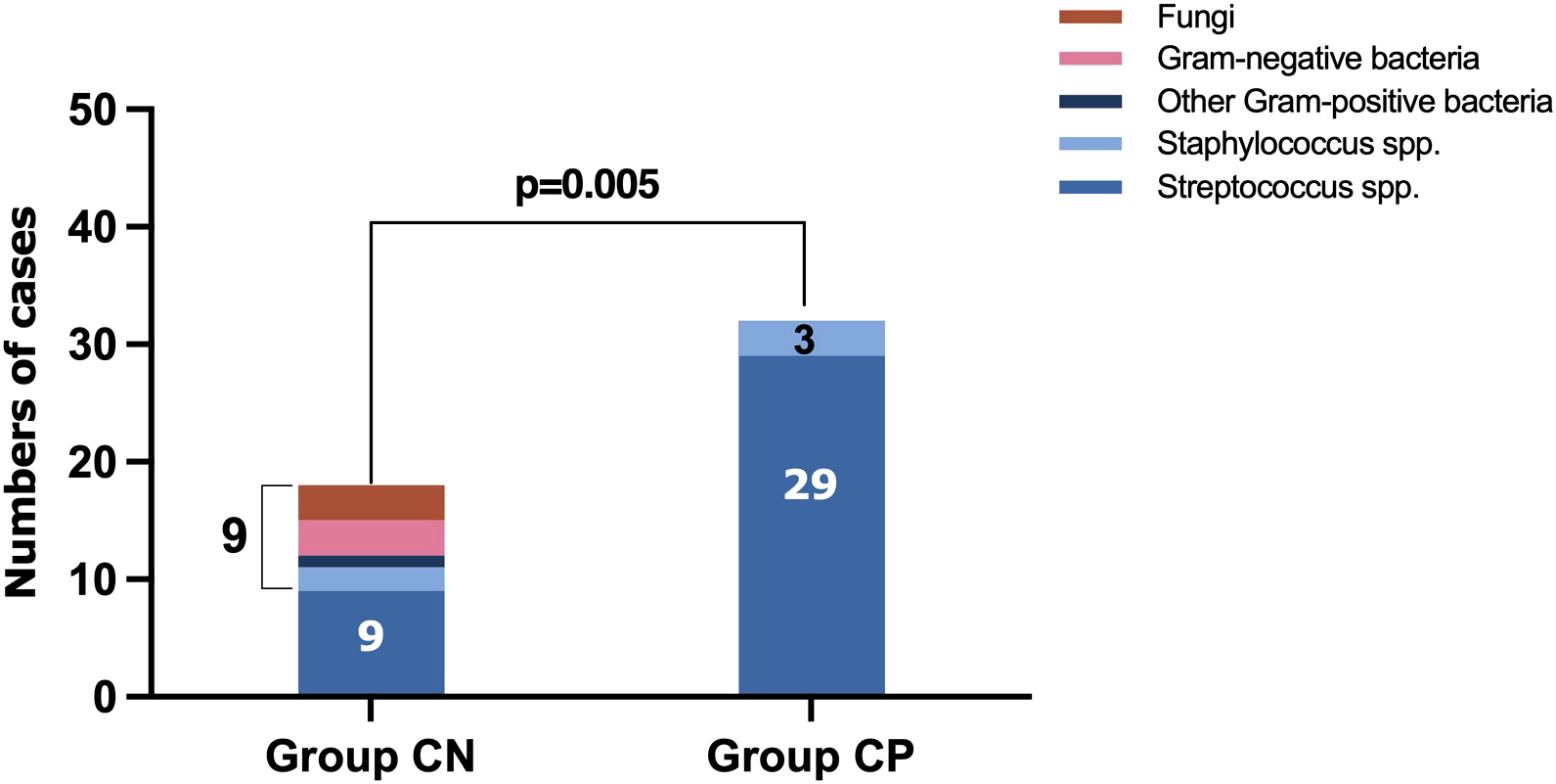
The main types of microbes in culture-negative IE and culture-positive IE patients. IE, infective endocarditis.

The mNGS results of culture-positive IE patients were very much consistent with the blood culture but not with the valve culture due to germ-adapted preoperative antibiotherapy. The most popular bacteria detected from culture-positive IE patients was *Streptococcus* spp. (29/90.6%, **Figure 2** and **Figure 3**), and the most frequently infected *Streptococcus* spp. were *Streptococcus gordonii* (9/28.1%, **Figure 2**). In addition to *Streptococcus* spp., pathogens detected by blood mNGS in culture positive IE patients were *Staphylococcus* spp. (2 cases of *Staphylococcus aureus* and 1 case of *Staphylococcus hominis*).

Different from blood culture-positive IE, the pathogens detected by mNGS in blood culture-negative IE patients appeared many other types of pathogens (1 case of *Staphylococcus aureus*, 1 *Staphylococcus epidermidis*, 1 *Parvimonas micra*, 1 *Klebsiella pneumoniae*, 1 *Paenibacillus elgii*, 1 *Stenotrophomonas maltophilia*, 1 *Candida tropicalis*, 1 *Candida albicans*, and 1 *Candida glabrata*, **Figure 1**), although *Streptococcus* spp. (9/50%, **Figure 2** and **Figure 3**) accounted for a larger proportion. Blood culture-negative IE patients had a greater rate of detection of non-streptococcal infection (50% *vs*. 9.4%, p=0.005, **Figure 3**).

### Adjustment of antibiotic regimens in culture-negative IE patients

In the CN group, There were 12 cases of culture-negative IE patients who have used antibiotics before admission. After the result of blood mNGS, 10 patients have changed their antibiotic regimens (**Table 4**), and most of them were non-streptococcal infection (6/10). Among them, anti-fungal infection agent such as voriconazole was added to patients with fungal infections (Case 6, 8, 13; **Table 4**). It is worth mentioning that case 15, with negative blood culture, was tested for Streptococcus oralis infection by mNGS and according to the test results, the antibiotic was downgraded and changed from vancomycin to penicillin (**Table 4**).

**Table 4:** Blood mNGS optimizing antibiotic treatment in culture-negative IE patients.

Case Number	Before mNGS test	After mNGS test
**Case 3**	Piperacillin/Tazobactam	Levofloxacin
**Case 4**	Penicillin+Levofloxacin	Telicoplanin+Imipenem/Cilastatin
**Case 5**	Mezlocillin/Sulbactam+Levofloxacin	Telicoplanin+Imipenem/Cilastatin
**Case 6**	Ceftriaxone+Levofloxacin	Voriconazole+Imipenem/Cilastatin+Linezolid
**Case 8**	Cefperazone/Sulbactam	Voriconazole
**Case 11**	Piperacillin/Tazobactam+Meropenem	Penicillin+Linezolid
**Case 13**	Moxifloxacin	Piperacillin/Tazobactam+Voriconazole
**Case 14**	Latamoxef	Piperacillin/Tazobactam
**Case 15**	Vancomycin	Penicillin
**Case 18**	Cefperazone/Sulbactam+Gentamicin	Vancomycin

mNGS, metagenomic next-generation sequencing; IE, infective endocarditis.

## Discussion

In the present study, we assumed that mNGS has a definite effect in the etiologic diagnosis of IE patients with positive and negative blood culture results, and may contribute to guiding targeted antibiotic treatment in IE patients.

The corner stone of IE diagnosis and therapy is pathogen identification, and effective antibiotic therapy is key to improving outcomes in IE patients regardless of the surgical valve procedure (Subedi et al., 2017; Shah et al., 2020). The modified Duke criteria and the 2015 ESC Guidelines recommended blood culture as the diagnostic standard (Li et al., 2000; Habib et al., 2015), but blood culture in IE has several disadvantages. First of all, the time consuming of blood culture is long but the result is slow that it usually takes 5-7 days, and the time consuming of special pathogenic bacteria culture even more than 7 days. Then, antibiotic use prior to culture tends to influence the positive rate of blood culture and valve culture. In addition, bacteria of the HACEK group or nutritionally variant streptococci require particular conditions for the growth in the laboratory often result in false negative results from blood cultures in IE patients. The technology of mNGS detection can largely bridge the shortage of blood culture in pathogen identification. In fact, there have been many studies confirming that the sensitivity of mNGS is superior to blood cultures and valve cultures in terms of etiological diagnosis in IE patients (Cheng et al., 2019). The most obvious advantage of mNGS is efficiency, because the result of mNGS often comes out within 48 hours, greatly improving the efficiency of identifying the etiology and guiding the adjustment of antibiotics. Timely and effective anti-infective treatment can reduce vegetative erosion of valves that the IE patients could possibly avoid valve surgery. Secondly, although the majority of patients had already applied empirical antibiotic therapy prior to admission which potentially leading to sterile cultures, mNGS technology has the advantage of high-throughput and unbiased detection and thus is less affected by prior antibiotic use, may even guide clinical antibiotic selection by detecting death bacterial fragments (Gu et al., 2019; Li et al., 2021). This advantage of mNGS is also reflected in the detection of special pathogens and pathogenic bacteria that are difficult to culture due to the harsh growth conditions. Additionally, mNGS can more broadly screen all potential pathogens in clinical specimens, helping to identify unexpected or novel organisms. In addition to traditional blood culture, mNGS was superior to other non-culture based technologies, such as serological assays or 16S rRNA PCR (Kolb et al., 2019; Godfrey et al., 2020), in the pathogen diagnosis. A recent study has evaluated the yield of NGS added to a Sanger sequencing-based 16S rRNA PCR assay in clinical practice for diagnosis of bacterial infection, and found that the positivity of the test was improved substantially (Flurin et al., 2022).

Therefore, in this study we chose mNGS assay to clarify the etiological diagnosis of endocarditis, and considering that part of enrolled IE patient had not suffered valve surgery, we chose blood mNGS and blood culture to identify the pathogen. The results found that the consistency of mNGS and blood culture in identified pathogenic bacteria was well. Though there were 18 IE patients who showed negative blood culture results, application of mNGS detected pathogenic bacteria in all of them. Based on the above, we endorse the apply of mNGS in blood culture positive IE patients in parallel with blood culture and susceptibility testing to improve the efficiency of testing and finally improve outcomes of IE patients.

Blood culture negative IE has long been a difficult problem to address in clinical and can occur in up to 60% (Shah et al., 2020). The reasons for a negative blood culture result in IE patient, in addition to the pathogen being not easily cultured or unculturable, are related to empirical antibiotic use when the fever is of unknown origin in the initial stage of the disease, leading to inhibition or killing of bacteria in the circulation (Gu et al., 2021). In our study, it was found that two-thirds of blood culture-negative patients had been treated with different kinds of antibiotics for symptoms such as fever before admission. Clearly, mNGS technology has greater advantages in blood culture-negative IE patients, suggesting that mNGS technology is suitable for application to pathogen detection in blood culture negative patients with a high suspicion of IE. In our present study, streptococcus and staphylococcus were the most common causative organism both in the culture-negative and culture-positive patients. Besides, 3 cases of fungi, 1 case of *Parvimonas micra*, 1 case of *Parvimonas micra*, 1 case of *Klebsiella Pneumoniae*,1 case of *Paenibacillus elgii*, and 1 case of *Stenotrophomonas maltophilia* were detected by blood mNGS from culture-negative IE patients. Rapid and precise etiological diagnosis can directly improve the formulation of clinical antibiotic protocols, and more studies are warranted to reveal the impact of mNGS results on the prognosis of IE patients, which was also the aim of this study.

The pathogenic bacteria in this study were predominantly streptococci, which is different from the phenomenon of predominant staphylococcal infection in developed countries and may be related to the underlying disease in IE patients (Shah et al., 2020). The proportion of IE patients with non-streptococcal infection who had negative blood cultures was higher than that with positive blood cultures, implying that empirical antibiotics are not suitable for IE patients with negative blood cultures. Among the 12 blood culture-negative patients for empirical antibiotic use included in our study, 10 of them had their antibiotic regimen adjusted after the mNGS results were reported, and 1 patient even had an antibiotic regimen downgraded. This result suggested that mNGS reduced unnecessary broad-spectrum antibiotic use on the basis of guiding targeted antibiotic therapy. In addition, some scholars have studied the relationship between inflammatory indicators and mortality in IE infected with *Staphylococcus aureus*, and found that the levels of PCT and mortality were closely related (Tascini et al., 2020), while in this study, although we found that the levels of PCT in blood culture-negative IE patients was higher than that in culture-positive cases, we did not find that PCT was associated with poor prognosis, and further studies on inflammatory indicators will be done in the future.

There were limitations in this study. First, the important limitation is that the current study is based on mNGS, a method known to deal with a relatively weak signal of pathogenic microorganisms as opposed to the most recent pathogen targeted sequencing technology. Not only this may have played a role in the inconsistency observed between mNGS results and blood cultures in the CP group but this also may have introduced a potential bias in the mNGS results of the CN group. Then, valve mNGS testing was not employed in this study because a subset of patients did not suffer valve surgery. Finally, this study was a cohort study and had a small sample size. Further studies with larger sample sizes are warranted to validate our observations.

## Conclusion

In conclusion, our study endorsed that blood mNGS as the method of first choice for the etiologic diagnosis of IE, especially in patients with negative blood cultures, and mNGS testing should be performed as early as possible to detect the etiologic pathogen, thereby optimizing anti-infective treatment regimens.

## Data availability statement

The data presented in the study are deposited in the EMBL repository, accession number PRJEB57970.

## Ethics statement

The studies involving human participants were reviewed and approved by the Ethics Committee of the First Affiliated Hospital of Zhengzhou University. The patients/participants provided their written informed consent to participate in this study.

## Author contributions

S-LL and XZ: software, formal analysis, data curation, and writing-original draft. XZ and J-ZT: conceptualization, methodology, and writing - review and editing. Z-ZY: validation and formal analysis. X-YZ: term, resources, project administration, and funding acquisition. All authors contributed to the article and approved the submitted version.
